# Masking Effect of Radiopharmaceutical Dose Extravasation During Injection on Myocardial Perfusion Defects During SPECT Myocardial Perfusion Imaging: A Potential Source of False Negative Result

**DOI:** 10.4274/mirt.88942

**Published:** 2018-10-09

**Authors:** Mohsen Qutbi

**Affiliations:** 1 Department of Nuclear Medicine, Taleghani Educational Hospital, School of Medicine, Shahid Beheshti University of Medical Sciences, Tehran, Iran

**Keywords:** Masking effect, radiopharmaceutical dose extravasation, myocardial perfusion defect, SPECT, false negative

## Abstract

Proper interpretation of SPECT myocardial perfusion imaging (MPI) is primarily based on strict adherence to standard procedural protocols from patient preparation to image processing and display. Inadvertent faulty injection of the radiopharmaceutical and, consequently, dose extravasation during SPECT MPI is a more important issue than that in any other diagnostic scintigraphic procedure. As it can be considered as a major source of false negative result, clinician’s awareness of this problem during interpretation is of great importance. In some occasions, no local clinical signs or image findings may be available to the interpreter to be aware of dose extravasation to adopt a suitable approach. Herein, we present a case with dose extravasation during stress phase, which is repeated another day with the same protocol, and the potential effects of dose extravasation on SPECT myocardial perfusion images from different aspects and useful image findings as hints are provided.

## Abstract (tr)

SPECT miyokard perfüzyon görüntülemenin (MPI) doğru yorumlanması hasta hazırlığından görüntü işlenmesine ve gösterilmesine kadar her aşamada standart protokollere katı bir şekilde uyulmasına bağlıdır. SPECT MPI’de radyofarmasötiğin istemsiz olarak hatalı enjeksiyonu ve sonuç olarak doz ekstravazasyonu diğer herhangi bir tanısal sintigrafik işlemde olduğundan daha önemli bir konudur. Yanlış negatif sonucun ana kaynaklarından biri olarak değerlendirildiğinden, yorumlanma sırasında klinisyenin bu sorunun farkında olması büyük önem taşır. Bazı durumlarda, doz ekstravazasyonunun farkında olunmasını sağlayacak lokal belirtiler veya görüntüleme bulguları olmayabileceğinden yorum sırasında bu duruma uygun bir yaklaşım fırsatı olmayabilir. Burada stres fazında doz ekstravazasyonu olan bir hasta sunulmaktadır, görüntüleme aynı protokolle başka bir gün tekrarlanmıştır. Bu bulgular doğrultusunda doz ekstravazasyonunun SPECT MPI üzerinde olası etkileri farklı açılardan ele alınmış ve yararlı görüntüleme bulguları belirtilmiştir.

## Figures and Tables

**Figure 1:**
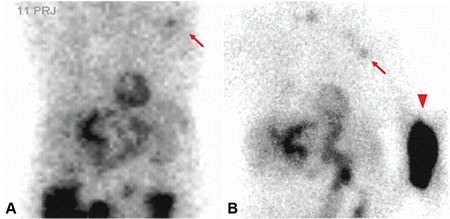
An 84-year-old female with a long history of asthma presented with an episode of chest pain and severe hypertension. The patient denied coronary angiography. Thus, a SPECT myocardial perfusion imaging (MPI) with dobutamine protocol was performed. Anterior projection of the raw cinematic image of stress SPECT MPI study (A) revealed a faint focal uptake in the region of left axilla (shown by arrow) as well as noticeably poor count statistics. In order to confirm the presence of tracer extravasation in the injection site, a planar anterior image with arms by the side (B) was obtained. As can be seen in B, considerable dose extravasation in the left forearm (shown by arrowhead) as well as a faintly hot axillary node ipsilateral to injection site were noted.

**Figure 2:**
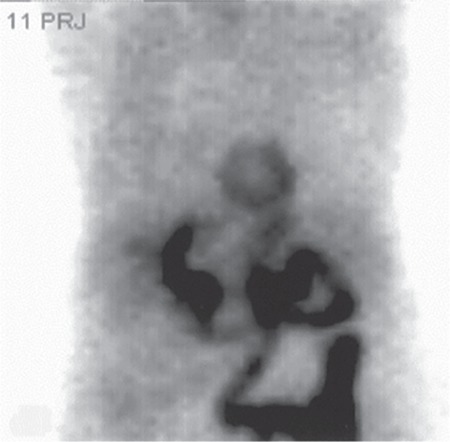
Anterior projection of the raw cinematic image of repeated stress study with the same protocol two days later demonstrated acceptable count statistics of the images without evidence of dose extravasation.

**Figure 3:**
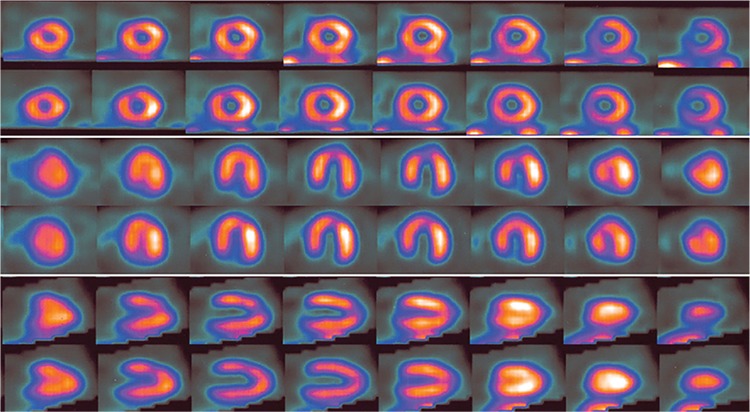
SPECT MPI of the initial study (upper row in each panel) and repeated study (lower row in each panel) showed a uniform tracer distribution in the initial study, but a mild perfusion defect in anterior and septal regions of left ventricular (LV) myocardium in the repeat study. From a technical viewpoint, the radiopharmaceutical with sufficient dose must be injected intravenously at peak stress during exercise or at target heart rate achieved by Dobutamine infusion. An injection with partly extravasated dose into the subcutaneous space effects the result, at least, in two possible ways. First, the amount of radioactivity entering into the circulation and then accumulating in the myocardium is insufficient that may cause a higher degree of noise in SPECT images (1). Second, which is even more troublesome, the extravasated dose gradually seeps out of the subcutaneous tissue into the circulation and then accumulates in the myocardium in the post-stress or resting condition. Therefore, the perfusion defects developing during peak stress may be attenuated or thoroughly masked (2). Moreover, the latter leads to a constantly high level of radioactivity in the background tissues. The added background counting rate and resultant higher scatter radiation are among the main factors of reducing contrast (i.e., myocardium-to-background ratio and defect-to-normal myocardium ratio). The added noise or decreased image information density as a result of lower radioactivity taken up by the myocardium contributes to impediments to visibility of defects, especially low-contrast defects (or mild perfusion defects) (3). As this issue may cause false negative interpretation and necessitates repeat of stress phase, the image should be carefully inspected for any evidence of extravasation. Although poor-count status (or grainy appearance) of the projection images and clumping of radioactivity in the myocardium (i.e., “sausage-string” pattern of LV walls) in tomographic slices (4) are considered as useful hints for dose extravasation, they are not invariably present and depend on the degree of extravasation. In patients with lower amount of extravasation, the decreased image count density might not be noticeable. Delayed images may show even better count statistics as a result of slow absorption of extravasated radioactivity (5). Another finding that implies dose extravasation is the visualization of hot axillary node ipsilateral to the injection site (6). But this is not a flawless way to discover extravasation. In some occasions, the axillary region may be out of the field-of-view and in other occasions, the node may be too faint to be readily visible. An easier and more certain way to realize possible extravasation is checking the injection site before imaging to avoid incorrect interpretation and repeating of the stress phase may be advisable.

## References

[1] Taillefer R (2016). The clinical importance of accurate measurement of injected doses for radionuclide myocardial perfusion imaging. J Nucl Cardiol.

[2] Travin MI (2015). Pitfalls and Limitations of Radionuclide and Hybrid Cardiac Imaging. Semin Nucl Med.

[3] Image quality in nuclear medicine (2012). In: Cherry SR, Sorenson JA, Phelps ME (eds). Physics in nuclear medicine, Philadelphia, Elsevier Saunders,.

[4] Depuey EG (2012). Image artifacts. In: Iskandrian AE, (ed). Atlas of nuclear cardiology: imaging companion to Braunwald’s heart disease. Philadelphia, Elsevier Saunders,.

[5] Strauss HW, Miller DD, Wittry MD, Cerqueira MD, Garcia EV, Iskandrian AS, Schelbert HR, Wackers FJ (1998). Procedure guideline for myocardial perfusion imaging. Society of Nuclear Medicine. J Nucl Med.

[6] Shih WJ, Collins J, Kiefer V (2001). Visualization in the ipsilateral lymph nodes secondary to extravasation of a bone-imaging agent in the left hand: a case report. J Nucl Med Technol.

